# Single-Stage Dermal Matrix and Skin Grafting to Treat a Complicated Hand Wound

**Published:** 2016-06-01

**Authors:** Mitchell Lyons, Alexis L. Parcells, Mark S. Granick

**Affiliations:** Division of Plastic Surgery, Rutgers New Jersey Medical School, Newark

**Keywords:** dermal matrix, reconstruction, skin grafts, Integra, bovine dermal matrix

## DESCRIPTION

A 58-year-old woman on immunosuppression therapy for a kidney transplant developed a paronychia refractory to antifungal or bedside debridement therapy. The infection rapidly progressed and required extensive operative debridement. Her resulting full-thickness dorsal hand defect measured 120 cm^2^ with exposed extensor tendons.

## QUESTIONS

1. **Describe the wound/defect seen and generate a differential diagnosis.**
2. **What are the goals for reconstruction in this patient?**
3. **What is Integra and what is it indicated for?**
4. **What are some causes of graft failure?**

## DISCUSSION

**Figure 1:**
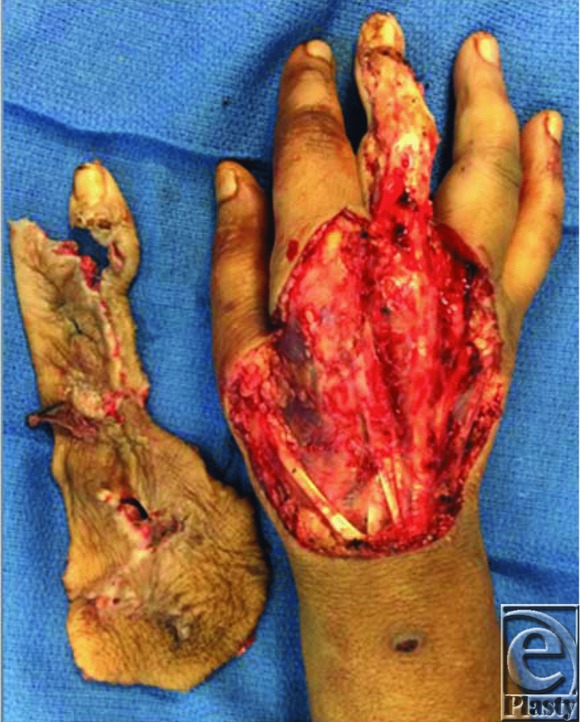
Dorsal hand wound after debridement of infection.

The wound seen in Figure 1 is the result of extensive operative debridement of the right hand due to a complicated paronychial infection refractory to conservative treatment. Paronychial infections are common and generally remain localized to the nail fold. If left untreated, they may progress into an abscess and spread proximally toward the palm. Differential diagnoses include felon, necrotizing or opportunistic infection, or malignant lesion.

Treatment of paronychia is centered on drainage and antibiotic therapy.^1^ If presenting in a delayed fashion or in an immunocompromised patient, extensive debridement may be required along with appropriate antibiotic or antifungal medication. After adequate debridement, reconstruction should focus on preserving hand function, specifically range of motion and tendon gliding. Skin graft alone may result in contracture and eventual stiffness.^2^ Regenerative advanced therapies have been shown to create a dermal layer to protect the underlying tendons and encourage dermal regeneration.

Integra (Life Sciences, Plainsboro, NJ) is a porous biological matrix composed of a cross-linked bovine tendon collagen and glycosaminoglycan scaffold that assimilates into wounds and stimulates vascularization and dermal regeneration. In the bilayer product, the matrix is covered with a layer of silicone that detaches as the matrix incorporates. Bilayer Integra is indicated for the management of partial- and full-thickness wounds and ulcers. Single-layer Integra is stripped of its silicone layer to allow for immediate thin skin autografting in a single-stage procedure.

Risk factors for graft failure include poor vascularization, fluid accumulation in the form of a seroma or hematoma, shearing, and infection.^3^ Minimizing graft failure is accomplished by ensuring a clean wound and by firmly stabilizing the graft until it is incorporated.

**Figure 2:**
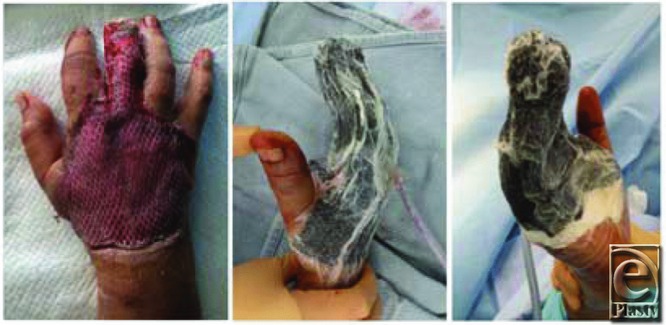
Dorsal hand wound after placement of dermal matrix, split-thickness skin graft, and negative pressure therapy.

In our patient, after adequate debridement and commencement of systemic antibiotic therapy, we applied a single-layer Integra matrix in combination with a split-thickness skin graft over the dorsal hand wound. Splinting the hand after application of Integra and a skin graft is essential to prevent shear and ensure successful integration of the graft. Although negative pressure therapy is not essential, it may improve graft survival, as it acts as a splint and prevents shearing or fluid accumulation (Fig 2) under the graft. This 1-step surgical procedure resulted in dermal regeneration and successful epithelialization.

**Figure 3:**
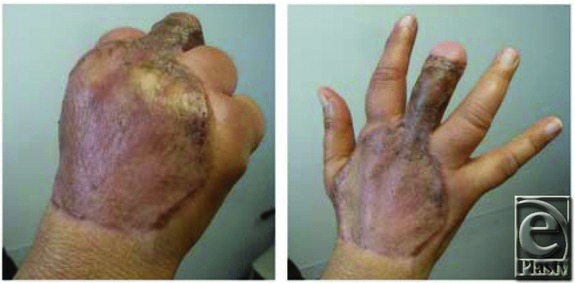
Healed wound demonstrating full range of motion.

Our patient's complete wound healing allowed her to work with occupational therapy and achieve good hand function (Fig 3). Upper limb reconstruction of large areas with exposed tendons and nerves frequently requires several surgical procedures. In contrast to current practice for skin substitutes, a “one-and-done” procedure can reduce the overall cost of treatment and decrease hospital stay all while providing excellent results and decreased morbidity.^4^ Our experience shows single-layer collagen-glycosaminoglycan matrix combined with skin grafting to be an effective method in the management of complicated hand wounds in selected cases.
